# EC313-a tissue selective SPRM reduces the growth and proliferation of uterine fibroids in a human uterine fibroid tissue xenograft model

**DOI:** 10.1038/s41598-019-53467-w

**Published:** 2019-11-21

**Authors:** Hareesh B. Nair, Bindu Santhamma, Kalarickal V. Dileep, Peter Binkley, Kirk Acosta, Kam Y. J. Zhang, Robert Schenken, Klaus Nickisch

**Affiliations:** 1 Evestra, Inc, 14805 Omicron Drive, San Antonio, TX 78245 USA; 2 Laboratory for Structural Bioinformatics, Center for Biosystems Dynamics Research, RIKEN, Yokohama, Kanagawa 230-0045 Japan; 3 Department of Obstetrics and Gynecology, University of Texas Health Science Center at San Antonio, 7703 Floyd Curl Drive, San Antonio, Texas 78229 USA

**Keywords:** Biochemistry, Drug discovery, Endocrinology

## Abstract

Uterine fibroids (UFs) are associated with irregular or excessive uterine bleeding, pelvic pain or pressure, or infertility. Ovarian steroid hormones support the growth and maintenance of UFs. Ulipristal acetate (UPA) a selective progesterone receptor (PR) modulator (SPRM) reduce the size of UFs, inhibit ovulation and lead to amenorrhea. Recent liver toxicity concerns with UPA, diminished enthusiasm for its use and reinstate the critical need for a safe, efficacious SPRM to treat UFs. In the current study, we evaluated the efficacy of new SPRM, EC313, for the treatment for UFs using a NOD-SCID mouse model. EC313 treatment resulted in a dose-dependent reduction in the fibroid xenograft weight (p < 0.01). Estradiol (E2) induced proliferation was blocked significantly in EC313-treated xenograft fibroids (p < 0.0001). Uterine weight was reduced by EC313 treatment compared to UPA treatment. ER and PR were reduced in EC313-treated groups compared to controls (p < 0.001) and UPA treatments (p < 0.01). UF specific desmin and collagen were markedly reduced with EC313 treatment. The partial PR agonism and no signs of unopposed estrogenicity makes EC313 a candidate for the long-term treatment for UFs. Docking studies have provided a structure based explanation for the SPRM activity of EC313.

## Introduction

The unmet need for medical management of uterine fibroids (UFs) has led to the discovery of various novel agents in recent years. These includes GnRH agonist, antagonists and selective progesterone receptor modulators (SPRMs). Various SPRMs are found to be very effective in range of biological activity including contraception, preoperative treatment of uterine leiomyomas^1^. UFs are the most common benign tumors in reproductive age women, are asymptomatic in at least 50% of afflicted women, in others, they cause significant morbidity and affect quality of life^2^. Women with UFs have abnormal uterine bleeding, dysmenorrhea, pelvic pain unrelated to menstruation, as well as pressure symptoms such as bloating, urinary frequency and bowel disturbances^3^. Current options for the management of UFs include medical therapy, minimally invasive procedures (e.g. uterine artery embolization, ultrasonic ablation), and surgery.

**Figure 1:**
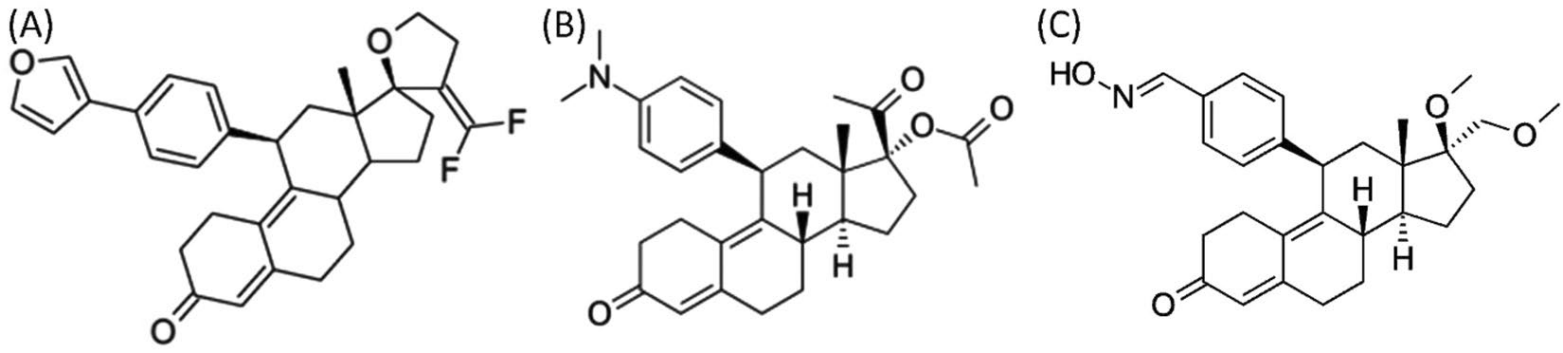
Chemical structures of (**A**) EC313, (**B**) ulipristal acetate and (**C**) asoprisnil.

UFs growth requires ovarian steroid hormones estradiol (E2) and progesterone (P4). Even though the endocrine support is from steroid hormones, interactions of sex steroids with growth factors cytokines were documented in UFs^4–7^. Recent interventions approaches have targeted inhibition of steroid hormones using antiestrogens/estrogen receptor modulators (SERMs) and SPRMs. Recent FDA-approved SPRMs, UPA and asoprisnil (ASO) (Fig. 1B,C) were withdrawn from the market due to the endometrial changes^8,9^. Another approach is gonadotropin-releasing hormone (GnRH) agonists, upon binding GnRH-analogues to GnRH receptors, an initial increase in the release of gonadotropins is followed by to GnRH receptor downregulation leads to reduced levels of sex hormones^10^. Often GnRH agonists are used as preoperative therapy^11,12^. GnRH agonists achieved decreasing menstrual bleeding and reducing fibroid volume by approximately 50%^13^. Adverse effects of GnRH agonist therapy involves the full range of menopausal symptoms including vasomotor symptoms and decreased bone mineral density^14^. The GnRH antagonists Elagolix was recently approved for endometriosis treatment and is expected to be submitted for market authorization for treatment of heavy menstrual bleeding associated with UFs^9,15^. Other antagonists such as relugolix and linzagolix are in development for the treatment of endometriosis and UFs. GnRH antagonists for UFs are often used in combination with estrogenic add-back medication to reduce the menopausal symptoms and loss of bone mineral density (BMD). Despite the concomitant add-back medication, the loss of BMD may be a major drawback for long-term treatment^1^. UFs often shows accumulation of extracellular matrix proteins and smooth muscle actin (α-SMA) in entwined bundles^16^. Collagenase treatment resulted a reduction of uterine fibroids from 5.3% to 2.4% in 96 hours^17^. It seems the structure of small fibroids differs considerably from large fibroids at cellular level, small fibroids are more cellular, whereas more vascularization was noted in large fibroids. It is also noted that a greater proportion of smooth muscle cells to fibroblasts is present in small fibroids^18^. Targeted agents that affect collagen or smooth muscle actin could be potential therapeutic agents for treating uterine fibroids.

EC313 is a steroidal progestin with differential binding towards steroids receptors such as PR and glucocorticoids (GR). Regarding E313’s binding affinity towards PR shows that it is <0.28% agonistic and 79.1% antagonistic when compared to R5020 and mifepristone respectively. EC313 binds to glucocorticoids minimally about 6.4% compared mifepristone and exert 100% antinidation (antiovulation/contraception) in rats. EC313 does not bind to ER. Also, we have shown that EC313 inhibits breast cancer cell proliferation and dichotomous branching of mammary glands in mice (44). Based on the previous studies we hypothesize that EC313 as an SPRM with preferential receptor binding capabilities, could be used to treat non-malignant estrogen driven gynecological conditions such as UFs. Here we assessed the potential therapeutic effect of EC313 (Fig. 1A) for the treatment of UFs. The efficacy of EC313 was tested in a patient derived UF xenograft in *NOD-SCID* mouse model and tissue markers of proliferation and fibrosis were analyzed by immunohistochemistry (IHC).

## Materials and Methods

### Materials

UPA, EC313, and ASO (Fig. 1) were synthesized by Evestra, Inc. release E2 pellets (0.05 mg/60day) were purchased from Innovative Research of America, Sarasota, FL. IHC was done at the University of Texas Health Science Center at San Antonio pathology core facility. Hydroxyl methyl cellulose was purchased from Sigma Aldrich, St. Louis, MO.

### Animal model

#### Animals

The *in vivo* study was approved by the Institutional Animal Care and Use Committee of the University of Texas Health Science Center at San Antonio (Protocol #20170019AP). The established murine model of transplantation of human UF tissue to immunodeficient NOD-SCID mice was used (1). Immunodeficient female, (NOD-SCID) mice were obtained from Charles River laboratories (USA), were housed according to institutional guidelines. Animals had the access to food and water ad libitum.

#### Collection of uterine fibroid tissue

All experimental protocols were approved by the institutional animal care and use committee of University of Texas Health Science Center at San Antonio (Protocol #20170019AP). Informed consent was obtained to collect human UF tissue. All experiments were performed in accordance with relevant regulation and guidelines of the University of Texas Health Science Center at San Antonio - Institutional Review Board (Protocol # HSC20070728H). The UF tissue used for current study is obtained from a single patient. The samples was transported to the laboratory on ice in Viaspan® buffer (Bristol –Myers Squibb) within 4 hours of collection. Tissue samples were cut aseptically into 2 × 2 × 2 mm sections and weighed. Equal weight grafts were used for transplantation.

#### Tissue transplantation

The mice were anesthetized using 1.5% isoflurane (4% for induction) in an oxygen/nitrous oxide 30%/70% mixture. Dorsal incisions were made, bilateral oophorectomy was performed, and wounds were stapled. Animals were monitored carefully during the recovery phase and 5 mg Rimadyl/kg bodyweight was administered s.c. for pain relief. Three weeks after ovariectomy, the UF tissue pieces, were transplanted s.c. using a trocar. During the same surgery, mice were supplemented s.c. with (60 day release estrogen) E2 pellets (Innovative Research of America, Sarasota, FL, USA). Animals received EC313 0.1 mg/kg, N = 6, or 1 mg/kg, N = 6), or UPA (5 mg/kg, N = 5) by subcutaneous injection (s.c) for five days per week for 8.5 weeks. E2 control animals received E2 + vehicle (N = 5). 0.2% hydroxymethyl cellulose (Sigma Aldrich, St. Louis, MO) in phosphate buffered saline (Life technologies, Carlsbad, CA) was used as vehicle. At termination of the study animals were weighed, sacrificed, and the uterus and fibroid xenografts were excised and weighed. Tissues were harvested, fixed in 10% formalin, and processed for immunohistochemistry (IHC) for ER, PR, Ki67, desmin and α-SMA.

### Molecular docking studies

To understand the possible agonistic and antagonistic activities of EC313, two stages of molecular docking studies have been performed. In stage-1 docking, we mainly explored the binding affinity of EC313 towards PR and compared with that of ASO and UPA (widely studied SPRMs). While in stage-2 docking, we investigated the affinities of four co-regulators (two co-activators: SRC1 and AIB1 and two co-repressors: SMRT and NCoR) towards the PR-SPRM complexes obtained from the stage-1 docking and compared with that of PR-P4 and PR-mifepristone complexes.

According to the previous studies, the ligand-bound PR interacts with DNA at P4 response elements to activate or repress the transcription activity through the recruitment of coregulatory proteins^19–22^. The binding of agonists or antagonists to PR induces conformational switching to the helix-12, which is a determining factor for the recruitment of co-activators or co-repressors. Molecules such as SPRMs exhibited mixed profiles of action, i.e., inducing decreased transcriptional activity compared with full agonists and increased transcriptional activity compared with full antagonists^23–26^. Studies postulated that SPRMs can induce an intermediate state of interactions between receptor and co-modulators^27–29^. These molecules partly stabilize at the agonist bound conformation of the receptor^30–34^ and allow co-activators to bind but with less efficacy than full agonists. The crystal structure of one of the well characterized SPRM ASO in complex with PR is reported in both agonist and antagonist bound conformations^35,36^. In the agonist bound conformation, the positioning of helix-12 is close to the ligand binding domain (LBD) and facilitates a binding site for co-activator proteins, whereas in the antagonist bound conformation, the helix-12 is positioned away from the LBD. These crystal structures (PDB IDs: 4a2j and 2ovh^35,36^) were used for the stage-1 docking studies. We have used an induced fit docking (IFD) protocol (Schrödinger LLC), to assess the binding of ligands towards the LBD. IFD predicts the ligand binding modes in a realistic way by applying concomitant structural changes in the receptor. No co-regulators were included in the receptor structure during stage-1 docking to avoid the conformational restrictions of the side chains of PR residues especially at the LBD upon ligand binding. The binding of ASO and UPA were also investigated in the same way. The corresponding poses obtained from docking studies were used for the MM-GBSA calculations and their binding energies were compared between the SPRMs. The detailed docking protocols including the protein and ligand preparations were explained in the supplementary text.

In stage-2 docking, the binding modes and affinities of four co-regulators towards PR-SPRM complexes were predicted. Three dimensional structures of all these co-regulators were extracted from different PDB entries and used for the docking studies (details shown in Supplementary Table 1). The co-activators and co-repressors were docked against agonist and antagonist bound conformations respectively using Hex 8.0.0^37,38^ and subsequently energy minimized using Maestro (Schrödinger LLC). The affinities of co-activators towards PR-P4 and affinities of co-repressors towards PR-mifepristone complexes were also calculated separately and compared with that of PR-SPRM complexes. The binding sites (grid) for co-regulators were defined on the PR based on the available literature information. The co-regulators were allowed only to probe at a defined grid space. After the docking, the best pose was selected based on the docking energy and was used for the MM-GBSA calculations followed by per-residue decomposition energy analyses.

### Statistical analysis

Quantitative data are expressed as the mean ± standard deviation and statistical significance was determined by Student’s *t* test.

## Results

**Figure 2:**
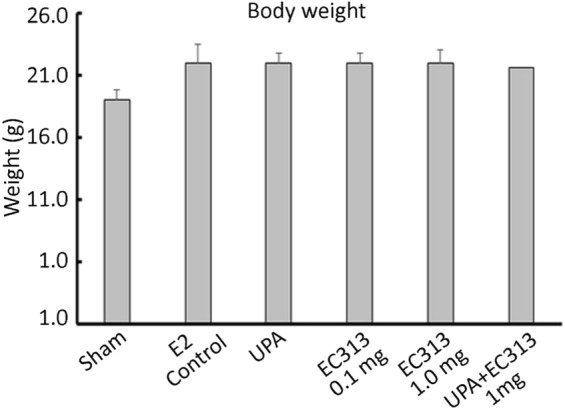
Body weight of the animals control/treated with test compounds. No significant changes in the body weight was observed between groups of experimental animals.

**Figure 3:**
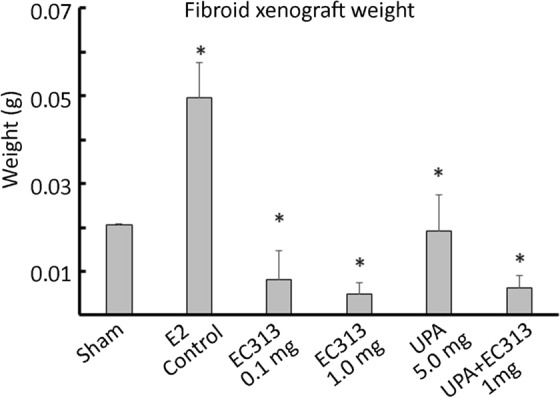
Human fibroids (PDX) are grown subcutaneously in immunodeficient NOD-SCID mice and treated with above compounds for 60 days (5 days/week). An optimum dose of E2 release (60 day pellet 0.05 mg/60 day release) supported growth of the subcutaneous grafts (n = 6). *p < 0.001 (E2-control vs. treatment groups).

**Figure 4:**
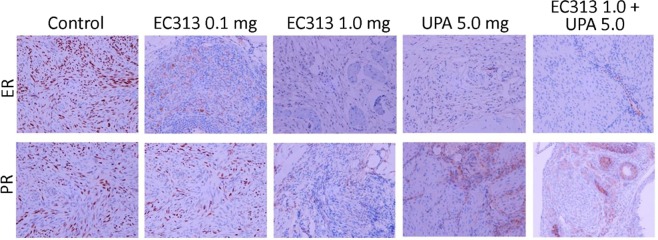
EC313 treatment reduced the levels of immunoreactive ER and PR levels required to stop fibroid growth

**Figure 5:**
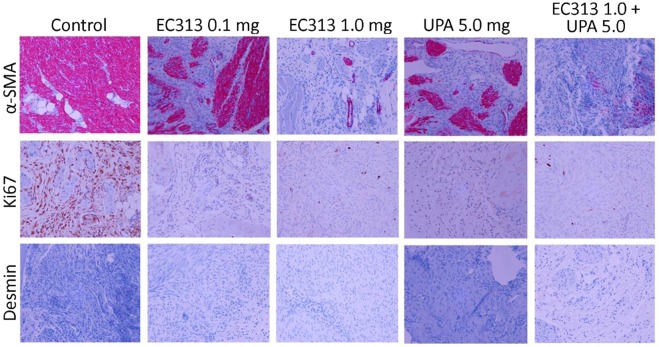
EC313 treatment reduced the proliferation of uterine fibroid cells indicated in Ki67 staining and inhibited the smooth muscle cells proliferation of the fibroid xenografts that express alpha- smooth muscle actin (α-SMA) and desmin as indicative markers of increased extracellular matrix activity.

In this study we have demonstrated an *in vivo* xenograft model of human UF in immunodeficient NOD-SCID mice. The optimum dose of E2 was required to support the growth of subcutaneous grafts. E2 dose were selected based on previous studies^2^. The grafted tissues showed UF cellular characteristics such as collagen bundles and abundance of smooth muscle actin as observed by Fritsch *et al*.^2^. The levels of ERα and PR stay expressed in UF grafts after 60 days in mice. The graft weights at the termination of the experiment was the primary endpoint, while histology and IHC were as secondary endpoints. The treatment didn’t affect the body weight of the animals when compared to untreated control animals (Fig. 2). The UF weight in all treated groups were found reduced. Both 0.1 and 1 mg/kg of EC313 were statistically superior to control in reducing the fibroid weight (Fig. 3, Table S2) (p < 0.001). ER as a target gene of PR was found upregulated in IHC staining in E2 treated control grafts versus treated ones. We have not observed a significant change in frequency or intensity of PR staining among these grafts, however reduced ER staining was noticed among treatment groups (Fig. 4). Alpha-SMA, was prominent in non-treated animals when compared to EC313 and UPA treated groups (Fig. 5). ER as a target gene of PR was found upregulated in IHC staining in E2 treated control grafts versus treated ones. Ki67 was decreased in EC313 treated mice compared to controls. The strongest staining observed in fibroid grafts of control animals, correlated with the highest grafted tissue weight gain (Fig. 5). Desmin staining established that the grafts maintained the intermediate filament features of smooth muscle cells in both treated and untreated mice (Fig. 5) as reported by Fritsch *et al*.^2^.

**Figure 6:**
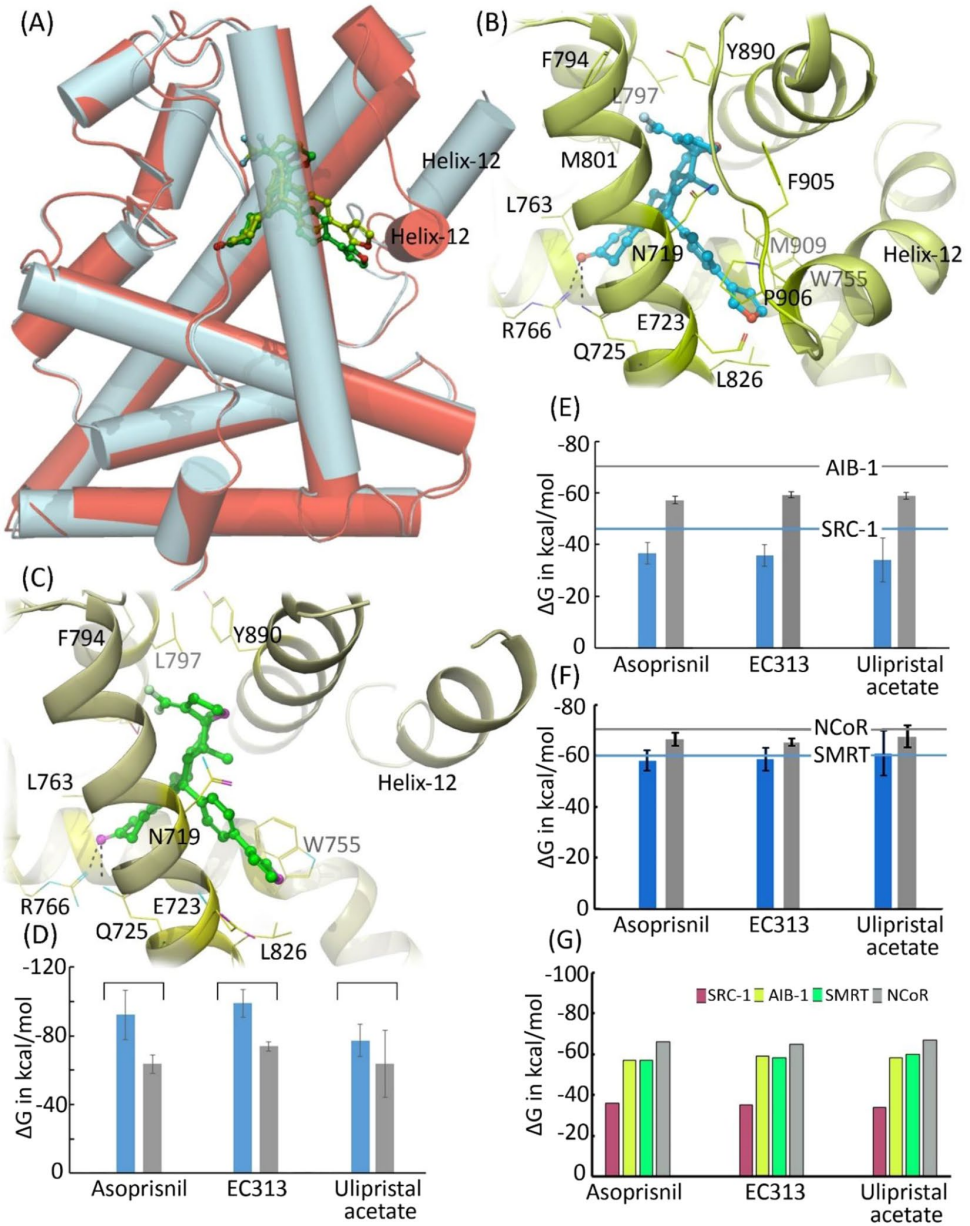
**(A)** Docking of EC313 to agonist (represented in red) and antagonist bound conformations (represented in grey). The differences in the position of helix-12 is obvious in both of these structures. The atomic level of interactions of EC313 towards agonist bound **(B)** and antagonist bound **(C)** conformations. The EC313 is represented in ball and stick model while the side chains of proteins are displayed with thin lines. **(D)** Binding energies of EC313 and two other control ligands (asoprisnil and ulipristal acetate) towards PR with agonist (represented with blue bars) and antagonist (represented with grey bars) bound conformations. **(E)** Binding energies of two selected co-activators, SRC-1 (represented with blue bars) and AIB-1 (represented with grey bars) towards PR-ligand complexes in the agonist bound form. The corresponding binding energies SRC-1 and AIB-1 towards a PR-progesterone complex are also marked in blue and grey lines. **(F)** Binding energies of two selected co-repressors, SMRT (represented with blue bars) and NCoR (represented with grey bars) towards PR-ligand complexes in the antagonist bound form. The corresponding binding energies SMRT and NCoR towards a PR-mifepristone complex are also marked in blue and grey lines. **(G)** Comparison of binding energies of co-regulators towards PR-SPRM complexes.

Our molecular docking studies of EC313 against agonist and antagonist bound conformations revealed a similar orientation for the ligand at LBD of PR (Fig. 6A). Further superimposition of PR-EC313 complexes (both agonist and antagonist bound form) has revealed a negligible structural deviation (~0.7 Å) for EC313 and shared a similar pattern of interactions. The orientation of steroid ring of EC313 is exactly same as other reported oxosteroid molecules.

### Stage-1 docking - docking towards agonist bound conformation

The ketone group located at the A-ring of EC313 anchors to R766 and Q725 through hydrogen bonds (Fig. 6B). The 17 α substitution in EC313 has oriented towards the hydrophobic sub-pocket (constituted by residues such as L715, L718, F794, L797, M801 and Y890) and favors a hydrophobic interaction. However, a halogen bond between one of the F atom of EC313 and the hydroxyl group of the Y890 was observed at a distance 3.2 Å, which were absent in ASO and UPA. The O atom in the oxolane of EC313 is oriented in such a way that it can readily accept a proton from the side chain of C891 and involved in a hydrogen bond. The phenyl furan ring of EC313 is involved in an edge to face stacking interactions with W755. In order to avoid the steric clashes and to facilitate the binding, side chains of M908 and M909 in helix-12 have adopted a different orientation compared to the ASO bound PR crystal structure. Apart from these interactions, several hydrophobic residues, especially L887, M756 and F778 and residues from helix-12, are involved in hydrophobic interactions with EC313.

Although the co-crystal structure of PR with ASO in the agonist bound conformation was available in the PDB, we repeated the docking calculations for ASO as a control experiment. The docking exactly reproduced the binding mode as in the crystal structure. However, a slight conformational change was observed for the amino acid side chains located at the LBD due to the ligand binding. The docking studies of UPA towards agonist bound conformation revealed a similar orientation for the oxosteroid ring as observed in EC313 and ASO. Hence, the hydrogen bonds between the ketone group of UPA and R766 and Q725 were maintained as in the previous cases. The MM-GBSA calculations suggested that EC313 exhibited an improved energy (−99 ± 14 kcal/mol) compared to ASO (−92 ± 8 kcal/mol) and UPA (−77 ± 7 kcal/mol) (Fig. 6D). Due to the substitution of bulky furan ring in EC313 compared to the N-hydroxymethanimine (in ASO) and N,N-dimethylmethanamine (in UPA) at the corresponding positions, more hydrophobic interactions with the side chains of helix 12 was observed for EC313 which may be partly contributed for the improved binding energy.

### Docking towards antagonist bound conformation

The binding mode of EC313 towards antagonist bound conformation was exactly the same as that of agonist bound conformation and hence almost similar type of interactions were produced by the ligand at the LBD (Fig. 6C). However, due to outward positioning of helix-12 in the antagonist bound conformations, no interactions were observed with the side chains of helix-12. Although the co-crystal structures of ASO and UPA in the antagonist bound conformations were available in the PDB, we repeated the docking studies as a control experiment. As expected, the bound poses were very similar to the crystal pose. Further, our MM-GBSA calculations revealed a significant reduction in the binding energies for all ligands in the antagonist bound conformations when compared to the agonist bound conformations. The difference in the energy might be due to the absence of interactions with the helix-12. The EC313 exhibited slightly improved binding energy (−73 ± 2 kcal/mol) towards PR compared to the asoprisnil (−63 ± 5 kcal/mol) and UPA (−63 ± 13 kcal/mol) (Fig. 6D).

### Stage-2 docking - docking of co-activators towards the agonist bound conformations

The docking studies of two co-activators revealed that SRC-1 binds to all of the PR-SPRM complexes with similar affinity (−36 ± 4 kcal/mol, −35 ± 4 kcal/mol and −34 ± 8 kcal/mol towards PR- ASO, PR-EC313 and PR-UPA complexes respectively) (Fig. 6E). However, compared to SRC-1, AIB-1 exhibited an improved binding affinity towards the PR-SPRM complexes (−57 ± 1 kcal/mol, −59 ± 1 kcal/mol and −58 ± 1 kcal/mol towards PR-ASO, PR-EC313 and PR-UPA complex respectively) (Fig. 6E). Furthermore, the binding of these co-regulators towards the PR-P4 complex revealed a remarkable difference in the affinities for both of the co-regulators. Both SRC-1 (−46 kcal/mol) and AIB-1 (−73 kcal/mol) exhibited improved binding affinity towards the PR-P4 complex. We further investigated the mode of interactions of these co-regulators towards PR-P4 complex to understand the reason for improved binding energies. When SRC-1 binds to the PR-P4 complex, the presence of a few salt bridges (between D745 (PR) and H6 (SRC1); E911 (PR) and H2 (SRC-1)) were noticed which were absent in the PR-SPRM complexes. Interestingly, the E911 located at the helix-12 had adopted a small rearrangement to facilitate a salt bridge interaction in PR-P4 complex. As discussed earlier, due to the bulkier size of the SPRMs and to accommodate them in the LBD, the side chains of M908 and M909 have rearranged. However, in the presence of a smaller sized agonist, these residues adopted different conformations in PR-P4 complex. The side chains of M908 and M909 are located at the vicinity of E911 and hence these conformational changes propagated a slight movement for E911 and facilitated the salt bridges. Due to the conformational rearrangement, several hydrophobic contacts were also observed between M908 and residues of SRC-1. At the same time, when AIB1 was docked to the PR-P4 complex, two unique salt bridges (between E907 (PR) and K3 (AIB-1); E911 (PR) and H2 (AIB-1)) were observed, which were absent in the PR-SPRM complexes. Several other hydrophobic interactions, especially with M908 and L727 were found to stabilize the interactions of AIB-1 with PR-P4 complex. As mentioned earlier, the side chains of M908 and M909 have adopted a similar conformation as observed in the PR-P4-SRC-1 complex. The role of M908 and M909 were reported previously to stabilize the agonist bound conformation of helix-12^35^. The result of our studies highlighted the importance of the conformational flexibility of residues at helix-12 in the determination of agonistic and partial agonistic activities.

The docking of two co-repressors, SMRT and NCoR, towards PR-SPRM complexes revealed that co-repressors have slightly better binding energies than co-activators, which may be due to the differences in the amino acid sequences between the co-regulators (Fig. 6F,G). It was reported that the SPRMs possess increased transcriptional activity compared with full antagonists^23–26,35^. The transcriptional repression activity is usually regulated via the binding of co-repressors. Based on this fact, we expected an improved binding affinity for the co-repressors towards PR-SPRM complex compared to the PR-antagonist complex. However, in our docking studies, both SMRT (−61 kcal/mol) and NCoR (−69 kcal/mol) exhibited better binding affinity towards the PR-mifepristone complex than PR-SPRM complexes (Fig. 6F). The affinities of SMRT and NCoR towards PR-ASO complex were −58 kcal/mol and −66 kcal/mol respectively. Similarly, the binding affinities of SMRT and NCoR towards PR-EC313 complexes were −53 kcal/mol and −65 kcal/mol respectively. The affinities of SMRT and NCoR towards PR-UPA were −60 kcal/mol and −67 kcal/mol respectively. Although the reason for improved antagonistic activities of EC313 compared to full antagonists remain unknown, our studies clearly explained partial agonistic activities of EC313.

## Discussion

UFs are often asymptomatic, 20–50% of women acknowledge menorrhagia, pregnancy loss, pelvic pressure and uterine pain^39^. A systematic review estimated total expenses for patients per year succeeding diagnosis or surgery to be $11,717 to $25,023^40^. Current pharmacological interventions including oral contraceptives, progestins, nonsteroidal anti-inflammatory drugs, antifibrinolytics, gonadotropin-releasing hormone agonists. Selective E2 or P4 receptor modulator progestins, danazol and aromatase inhibitors are in current clinical practice^41^.

SPRMs are small molecules binding with high affinity to the PR receptor binding pocket, modulating the activity of the receptor. SPRMs include a wide spectrum of substances ranging from highly potent receptor antagonists to compounds with a balanced mix of partial-agonistic and antagonistic effects such as UPA or vilaprisan and mesoprogestins like ASO. It is clear from our in silico molecular docking studies that EC313 exhibited higher binding energies towards PR when compared to ASO and UPA. In clinical trials, UPA as well as ASO showed significant decrease in uterine fibroid volumes after termination of treatment, collectively due to reduced cell proliferation, angiogenesis, extracellular matrix and enhanced apoptosis^42–46^. Previously we have demonstrated that EC313 induces proapoptotic genes and possesses antiproliferative effects in breast cancer cells^47^. Improved antiprogestational activity probably gives EC313 a higher rate of antiproliferative activity and proapoptotic effects. Previously documented studies show that antagonistic SPRMs including mifepristone, UPA and vilaprisan have progestagenic activity compared to mesoprogestins like ASO and EC313 and reduced PR agonism in animal models^48^. It is well accepted that uterine fibroid growth is dependent on E2 and P4^49^. More recent studies suggest that progesterone and the PR shows additional significant role than E2 in PR synthesis^27,46,50^. However, latest scientific thoughts point to the fact that different progestins interacts with ER differently^51^. This study states that, PR associates with ERα and modulate ERα chromatin binding events^51^. In our studies we have seen a similar effect with EC313 where it modulates ER^52^ and partially downregulates ER when compared to full PR antagonist (unpublished data). Based on the current study we believe that molecules such as EC313 that preferentially binds to PR in an agonistic mode slightly different than ASO and UPA helps to attain a favorable drug like characteristics. EC313 may effectively handle issues with estrogen induced complications (occult breast tumors, reduced bone mineral density and irregular bleeding profiles) and potentially minimize the necessity of estrogen add-back therapy. The differential effects of progestins on its agonistic versus antagonistic profiles on PR binding is not completely resolved yet. In the current study we have made an attempt to systematically compare 2 different SPRMs UPA and ASO with EC313 on its receptor bound structures in the presence and absence co-regulators using *in silico*. Our data explain differential PR binding effects and perhaps tissue selectively of EC313 over UPA and ASO.

### Summary

In summary, we have demonstrated that both E2 and P4 are essential for fibroid xenograft growth, and a novel mesoprogestin (EC313), exhibiting antiprogestational as well as mild PR agonistic activity. EC313 may be superior or comparable to current standard of care (SOC) such as UPA to abolish growth of uterine fibroids. As a novel SPRM of the mesoprogestin-type with tissue selectivity, EC313 has distinct pharmacologic advantageous of oral bioavailability and *in vivo* stability. Potentially based on the mix of PR agonistic and antagonistic activities, EC313 showed comparable efficacy to UPA in the UF mouse model. The partial PR agonistic activity and the lack of unopposed estrogenicity makes EC313 an interesting candidate for the continuing treatment for uterine fibroids. Even though the human patient derived xenograft (PDX) UF model recapitulates the disease morphologically and biochemically, further studies regarding the mutation status of recently noted genes inhuman uterine fibroids such as MED12 or HMGA2 is warranted. We are aware that this study is also limited due to the lack of components of the immune system and possible artefacts due to effects of the human-mouse microenvironment.

## Supplementary information

EC313-a tissue selective SPRM reduces the growth and proliferation of uterine fibroids in a human uterine fibroid tissue xenograft model
