# Supplementary material for: EC313-a tissue selective SPRM reduces the growth and proliferation of uterine fibroids in a human uterine fibroid tissue xenograft model
Source: Sci Rep. 2019 Nov 21;9:17279. doi: 10.1038/s41598-019-53467-w (PMC6872653; doi:10.1038/s41598-019-53467-w)
Supplement: Supplementary file 1 — EC313-a tissue selective SPRM reduces the growth and proliferation of uterine fibroids in a human uterine fibroid tissue xenograft model [file 41598_2019_53467_MOESM1_ESM.docx]

**EC313-a tissue selective SPRM reduces the growth and proliferation of uterine fibroids in a human uterine fibroid tissue xenograft model**

Hareesh B. Nair^1@^, Bindu Santhamma^1^, Kalarickal V. Dileep^2^, Peter Binkley^3^, Kirk Acosta^1^, Kam Y.J. Zhang^2^, Robert Schenken^3^, Klaus Nickisch^1^

*Evestra, Inc, 14508 Omicron Drive, San Antonio, TX 78245.

^ᵻ^Laboratory for Structural Bioinformatics, Center for Biosystems Dynamics Research, RIKEN, Yokohama, Kanagawa 230-0045, Japan.

^#^Department of Obstetrics and Gynecology, University of Texas Health Science Center at San Antonio, 7703 Floyd Curl Drive, San Antonio, Texas 78229.

**@Corresponding Author:**

Hareesh B. Nair, PhD., Evestra, Inc, 14508 Omicron Drive, San Antonio, TX 78245, USA; Email: [hnair@evestra.com](mailto:hnair@evestra.com), Ph: 210-278-9819

**Key words:** Uterine fibroids (UF), selective progesterone receptor modulator (SPRM), Ulipristal acetate (UPA) and EC313

**Supplementary information**

**Protein preparation**

Four receptor structures were used in the current studies. Among these receptor structures, PR-ASO complex (both in agonist and antagonist form with PDB IDs of 4a2j, 2ovh respectively)^1,2^ were used to probe the affinities of selected ligands such as ASO, EC313 and UPA. The structure of PR bound with progesterone (PDB ID: 1a28)^3^ was used to investigate the affinity of an agonist (progesterone) towards the receptor. Since the antagonist bound structure of PR was not available in the PDB, we modeled the PR-mifepristone complex (the modeling was done based on the PDB ID: 2w8y)^4^ and investigated the affinity of the mifepristone. Both of the progesterone and mifepristone bound structures were used as a positive controls in the current studies.

All of these selected protein structures were downloaded from the PDB and prepared for docking studies using protein preparation wizard in Maestro (Schrödinger LLC). During the protein preparation, the crystallographic water molecules were deleted, missing atoms and polar hydrogens were added. The prepared protein was further energy minimized by applying a cut off of 0.30 Å using OPLS 2005 force field.

**Ligand preparation**

All the selected ligands such as ASO, EC313, UPA, P4 and mifepristone were prepared using Ligprep module, Maestro (Schrödinger LLC) with MMFF force field. During preparation, the geometry of ligands were optimized by correcting the bond angles and bond lengths. Multiple conformations for the ligands were generated at pH 7.0 ± 2.0 and all these conformers were used for the docking studies.

**Induced fit docking**

In order to study the affinities of selected ligands towards the corresponding receptor structures, we used the induced fit docking module, Maestro (Schrödinger LLC). During this stage-1 docking, the ligand binding site on the PR was identified from the crystallographic ligand and the protein residues that are 6 Å away from the centroid of the ligand were made flexible. As a first step of induced fit docking, the Glide docking of the ligand was carried out using a softened potential. By default, 20 poses per ligand were generated. Further side-chain prediction followed by minimization for each protein-ligand complex were performed. A total of 20 poses was generated as a part of IFD calculations and each pose was subjected to MM-GBSA calculations. All of the MM-GBSA calculations were performed using VSGB solvation model with OPLS3 force field.

**Protein peptide docking**

To investigate the binding affinities of co-regulators towards PR-ligand complexes obtained from stage-1 docking, protein-peptide docking was performed. For this stage-2 docking, four co-regulators (two co-activators: SRC1 and AIB1 and two co-suppressors: SMRT and NcOR) extracted from various protein structures (Table S1) were used. All of the selected co-regulators were prepared using protein preparation wizard, Maestro (Schrödinger LLC) as discussed earlier. The number of amino acids in the co-activators were kept the same to avoid the additional energy contributions during the docking studies. Similarly, the number of residues in the co-repressors were also used as the same.

| **Sl No** | **Name of the co-regulator** | **Type** | **Sequence** | **No of amino acids** | **Extracted from** |
| --- | --- | --- | --- | --- | --- |
| 1 | SRC-1 | Co-activator | HKILHRLLQD | 10 | PDB ID: 3pcu^5^ |
| 2 | AIB-1 | Co-activator | HKKLLQLLTC | 10 | PDB ID: 3l3x^6^ |
| 3 | SMRT | Co-repressor | LEAIIRKALMG | 11 | PDB ID: 2ovh^2^ |
| 4 | NCoR | Co-repressor | LEDIIRKALMG | 11 | PDB ID: 2ovm^2^ |

S1: Details of the co-regulators used for docking studies

The binding of co-regulators towards the PR-ligand complexes was studied using Hex 8.0.0. The program used Spherical Polar Fourier (SPF) correlations rather than Fast Fourier Transform (FFT) based search to probe the potential binding modes of the co-regulators on the PR. In the SPF search, 5 rotational and 1 translational degree of freedom was present whereas in FFT based search 3 rotational and 3 translational degrees of freedom were there. Based on the shape complementarity excluded volume with an optimal in vacuo electrostatic contribution of the final docked poses with the best energy was retrieved.

**Energy Minimization**

The ‘macromodel’ module implemented in Maestro (Schrödinger LLC) with OPLS3 force field was used for the energy minimization of PR-ligand-coregulatory complexes. The default parameters, Polak-Ribier Conjugate Gradient method with maximum iterations of 2500 was used for the energy minimization. The output structure after energy minimization was further analyzed for any close contacts especially at the binding interface of PR. It was assumed that the energy minimization might have produced the structural changes that are similar to the structural changes imparted by the protein-peptide interactions in the real scenario.

**MM-GBSA calculations**

Prime MMGBSA DG bind of PR-ligand complexes and PR-ligand-coregulatory complexes were calculated using OPLS3 force field with a solvation model VSGB and a sampling method ‘minimize’. The binding free energy is calculated with the equation: ΔG (bind) = E_complex (minimized) - (E_ligand (minimized) + E_receptor (minimized))

| **Compound** | **Dose (mg/kg)** | **Body weight (g)** | **Fibroid weight (g)** | **Uterine weight (g)** |
| --- | --- | --- | --- | --- |
| E2 control  N=5 |  | \| 23.1 \| \| --- \| \| 22.5 \| \| 21.3 \| \| 21.8 \| \| 21.2 \| | \| 0.0812 \| \| --- \| \| 0.0836 \| \| 0.0226 \| \| 0.0369 \| \| 0.0232 \| \| 0.0802 \| \| 0.0812 \| \| 0.0204 \| \| 0.0359 \| \| 0.0241 \| | \| 0.0915 \| \| --- \| \| 0.1095 \| \| 0.0903 \| \| 0.0955 \| \| 0.0978 \| |
| UPA  N=5 | 5 | \| 24.0 \| \| --- \| \| 22.9 \| \| 21.4 \| \| 21.7 \| \| 20.0 \| | \| 0.0581 \| \| --- \| \| 0.0341 \| \| 0.0076 \| \| 0.0055 \| \| 0.0033 \| \| 0.0189 \| \| 0.0062 \| \| 0.0064  0.0146  0.0078 \| | \| 0.04705 \| \| --- \| \| 0.01938 \| \| 0.1191 \| \| 0.0672 \| \| 0.071 \| |
| EC313  N=6 | 0.1 | \| 22.9 \| \| --- \| \| 22.5 \| \| 22.7 \| \| 21.5 \| \| 20.8 \| \| 21.7 \| | \| 0.00367 \| \| --- \| \| 0.00358 \| \| 0.00388 \| \| 0.0077 \| \| 0.0104 \| \| 0.01036 \| \| 0.006 \| \| 0.0035 \| \| 0.0077 \| \| 0.0252 \| | \| 0.068 \| \| --- \| \| 0.0401 \| \| 0.0401 \| \| 0.0431 \| \| 0.0436 \| \| 0.0499 \| |
| EC313  N=6 | 1.0 | \| 23.2 \| \| --- \| \| 22.5 \| \| 21.1 \| \| 21.4 \| \| 21.9 \| \| 21.6 \| | \| 0.0085 \| \| --- \| \| 0.003 \| \| 0.004 \| \| 0.0046 \| \| 0.0069 \| \| 0.0069 \| \| 0.0023 \| \| 0.0073 \| \| 0.0044 \| \| 0.0011 \| | \| 0.0704 \| \| --- \| \| 0.0445 \| \| 0.0590 \| \| 0.0131 \| \| 0.0515 \| \| 0.0438 \| |
| EC313+UPA  N=5 | 1.0/5 | \| 21.0 \| \| --- \| \| 19.9 \| \| 21.2 \| \| 22.3 \| \| 22.6 \| \|  \| | \| 0.0047 \| \| --- \| \| 0.0046 \| \| 0.0035 \| \| 0.0043 \| \| 0.0046 \| \| 0.0063 \| \| 0.007 \| \| 0.0126 \| \| 0.0097 \| \| 0.0044 \| | \| \| 0.0522 \| \| --- \| \| 0.0349 \| \| 0.0648 \| \| 0.0824 \| \| 0.055 \| \| \| --- \| --- \| --- \| --- \| --- \| --- \| \|  \| \|  \| \|  \| \|  \| \|  \| |

S2: Test for anti-uterine fibroid activity of EC313: Individual data for body weights, fibroid weights and uterine weights. The test included inoculation of 3 individual samples of human uterine fibroids. At least two fibroids from each animal were measured at the end of study.

**Table legends**

**Table S1:** Details of the co-regulators used for docking studies.

**Table. S2:** Test for anti-uterine fibroid activity of EC313: Individual data for body weights, fibroid weights and uterine weights. The test included inoculation of 3 individual samples of human uterine fibroids. At least two fibroids from each animal were measured at the end of study.

**References**

1 Lusher, S. J. *et al.* X-ray structures of progesterone receptor ligand binding domain in its agonist state reveal differing mechanisms for mixed profiles of 11beta-substituted steroids. *J Biol Chem* **287**, 20333-20343, doi:10.1074/jbc.M111.308403 (2012).

2 Madauss, K. P. *et al.* A structural and in vitro characterization of asoprisnil: a selective progesterone receptor modulator. *Mol Endocrinol* **21**, 1066-1081, doi:10.1210/me.2006-0524 (2007).

3 Williams, S. P. & Sigler, P. B. Atomic structure of progesterone complexed with its receptor. *Nature* **393**, 392-396, doi:10.1038/30775 (1998).

4 Raaijmakers, H. C., Versteegh, J. E. & Uitdehaag, J. C. The X-ray structure of RU486 bound to the progesterone receptor in a destabilized agonistic conformation. *J Biol Chem* **284**, 19572-19579, doi:10.1074/jbc.M109.007872 (2009).

5 Zhang, Y. *et al.* (+)-Rutamarin as a dual inducer of both GLUT4 translocation and expression efficiently ameliorates glucose homeostasis in insulin-resistant mice. *PLoS One* **7**, e31811, doi:10.1371/journal.pone.0031811 (2012).

6 Zhou, X. E. *et al.* Identification of SRC3/AIB1 as a preferred coactivator for hormone-activated androgen receptor. *J Biol Chem* **285**, 9161-9171, doi:10.1074/jbc.M109.085779 (2010).
